# Propagation of Some Local Fig (*Ficus carica* L.) Cultivars by Hardwood Cuttings under the Field Conditions in Tunisia

**DOI:** 10.1155/2014/809450

**Published:** 2014-11-09

**Authors:** Fateh Aljane, Sabrine Nahdi

**Affiliations:** Laboratoire d'Aridoculture et Cultures Oasiennes, Institut des Régions Arides, 4119 Médenine, Tunisia

## Abstract

This research was carried out in Southeast of Tunisia in 2009 and 2010, in order to study the propagation of six (*Ficus carica* L.) cultivars by using hardwood cuttings under the field conditions. The effect of the cultivars and the type of buds, shoots age, shoots length, and shoots diameter were recorded. Ten cuttings per cultivar and/or cutting types with three replications were planted in rooting unit. Percentage of root emergence and six morphological parameters of young fig plants were measured. Results showed that the responses of cuttings as fig nursery plants presented a high variability among the five cultivars. The most widely varied characters were % root emergence (RE) and cumulative growth of young plant (CG). The first one ranged from 10% to 90%, the second varied within 32 and 112 cm. Concerning the ‘‘BITHER” cultivar, 6 cutting types with different age, length, and diameter were evaluated. Results showed a great variation in % of root emergence (0–90%), length of nursery plant (3–77 cm), and number of roots/nursery plant (0–29 roots). The present research showed that the hardwood cutting of local fig cultivars can be propagated under field conditions in Southeast of Tunisia.

## 1. Introduction

Fig (*Ficus carica* L.) is one of the traditional Mediterranean species. Fig fruit represents an important constituent of the diet, because of their nutritional and medicinal values [1]. This type of diet is considered one of the healthiest and is associated with longevity [2]. Figs are an excellent source of minerals, vitamins, polyphenols, and dietary fiber; they are fat and cholesterol-free and have a high antioxidant activity [3].

Figs have recently attracted a great deal of attention and are widespread throughout the world. The world produces over one million tons of figs yearly, of which 82% are produced in Mediterranean countries [4].

In Tunisia, the production is about 29 000 tons; it represents 3% of total world production [4]. The fig is an economically important fruit species which is cultivated extensively in Southeast regions [5]. Fig nursery plants production is mainly based on cuttings taken from the mother tree and placed in a rooting, which will eventually produce roots and shoots [6–8]. The traditional method for growing fig consisted in taking cuttings from one- or two-year-old shoots and directly planting them in soil to obtain new trees [6]. Furthermore, farmers have used layering method for propagating fig in orchards [9]. However, the investigation on ways to propagate fig tree material and produce vigorous and healthy fig plants under field conditions becomes necessary in order to develop fig sector.

The objective of this study was to characterize and compare fig nursery plants of some local fig cultivars by using hardwood cuttings under field conditions. In this way, five cultivars and six cuttings sizes were compared in order to optimize the propagation method of fig in Tunisia.

## 2. Material and Methods

### 2.1. Plant Material

In this research, Hardwood cuttings of 4 uniferous (one production per year) fig cultivars (“Bayoudhi,” “Jemâaoui,” “Ragoubi,” and “Zidi”), one biferous (two productions per year) (“Bither”), and one caprifig (male fig) (“Bouharrag”) were used as plant material. Two experiments were done in 2009 and repeated in 2010. Fig tree is a species having high level of adaptation to different soil types and dry conditions. These cultivars are frequently cultivated for producing fresh and sun drying fig in arid region of Tunisia (100–200 mm annual precipitation). These ones are widely grown in southern Tunisia and were described in a previous work [10, 11]. Hardwood cuttings were taken at the beginning of February 2009 and February 2010, from adult fig trees of the germplasm collection of Arid Land Institute (IRA)—Medenine, located in El Gordhab—Tataouine (Tunisia).

### 2.2. Experiments Description (Table 1)

**Experiment 1 .**

It consists in comparing the ability of cutting of 5 cultivars (“Bayoudhi,” “Jemâaoui,” “Ragoubi,” “Zidi,” and “Bouharrag”). The experiment was carried out with three replicates in a split-plot design [12]. Ten cuttings per replicate and per cultivar were used, with a total of 150 cuttings. Cuttings of one year of 30 cm length were used.

**Experiment 2 .**

It was involved on the cultivar “Bither” with 6 cuttings types as described below:

 Type 1: cuttings of one-year-old shoot, length (30 cm), and diameter (<1.5 cm);
 Type 2: cuttings of one-year-old shoot, length (40 cm), and diameter (<1.5 cm);
 Type 3: cuttings of one-year-old shoot, length (60 cm), and diameter (<1.5 cm);
 Type 4: cuttings of 2-year-old shoot, length (30 cm), and diameter (<1.5 cm);
 Type 5: cuttings of 2-year-old shoot, length (40 cm), and diameter (<1.5 cm);
 Type 6: cuttings of 2-year-old shoots, length (60 cm), and diameter (>2 cm).

This study was planned out with three replicates in a split-plot design [12]. Ten cuttings per replicate and per cutting types were used, with a total of 180 cuttings.

During this research, cuttings were planted directly in rooting units (2.5 × 0.65 m) under field conditions. The distance between cuttings is around 0.25 m on the same row [13]. Cuttings were planted in an inclined position (angle of 45°) on sandy soils, where 3/4 of cutting is sinking into the soil at the Professional Agricultural Training Center (CFPA) El Gordhab—Tataouine (Tunisia). All plants were irrigated by submersion technique with 40 l/rooting unit/15 days on the early morning during the course of the experiment in the three seasons. Maintenance works such as weed control and hoeing were carried out during the whole vegetation period. Organics and chemicals fertilizers were not used during the experiments.

### 2.3. Measured Parameters

Root emergence percentage, number of emerged buds/nursery plant, length of nursery plant from the soil surface, and diameter of nursery plant were measured on the section  5 cm higher than soil surface and number of leaves/nursery plant. At the drawing out of young plants, cumulative growth (cutting length + new growth) of nursery plant and number of roots/nursery plant were determined [12, 14]. All parameters were measured at the end of the vegetation period (almost 8 months after plantation). Emerged buds, leaves, and roots of each plant were counted manually. Band measuring device was used for the height measurements of each nursery plant. Stem diameter was measured with electronic caliper.

### 2.4. Statistical Analysis

Data were subjected to analysis of variance (ANOVA), using STATBOX version 6.40. In case of significant cultivars and cutting type's effects, comparison of values was performed by means of the least significant difference (LSD) test at a significance level of 0.05, as the percentage root emergence (RE %) was calculated by the means of three replications.

## 3. Results

### 3.1. Comparison of the Five Young Plants Fig Cultivars

The descriptive data analysis revealed a high variability among all the morphological characters of young plant fig cultivars studied under the field conditions. The most widely varied characters were root emergence percentage (10%–90%) and cumulative growth of nursery plant (32–112 cm). Considerable range of variation was also noticed in length of nursery plant (4 to 70 cm) and number of leaves/nursery plant; it varied from 3 leaves to 63 leaves, whereas very low variation was noted in number of emerged buds/nursery plant, diameter of nursery plant, and number of roots/nursery plant (Table 2).

The mean values of all morphological characteristics observed were statically different at a 5% level (Table 3). This indicates a certain variation within cultivars, especially for length, diameter of nursery plants, number of leaves, and cumulative growth of nursery plants. Root emergence percentage (% RE) had the highest values (65%) in “Jemaâoui” and “Rogabi” cultivars and the lowest values (15%) in “Bayoudhi.” Nursery plants characteristics like length of nursery plant (LP), diameter of nursery plant (DP), cumulative growth of nursery plant (AG), and number of roots/nursery plant (NR) exhibit the highest values in cultivars “Bayoudhi.” Finally, the number of leaves/nursery plant (NL) was significantly high in cultivars “Bouharrag” (37 leaves) and “Bayoudhi” (27 leaves).

The result of this part can provide that the cultivars choice is very important to produce sufficient amounts of vigorous nursery plants. Furthermore, “Bayoudhi” cultivar is very adapted to agroecological conditions, which can be eventually developed by farmers. Moreover, the length of nursery plant (LP), diameter of nursery plant (DP), and number of roots (NR) are very important quality characters for selection of nursery plants of fig cultivars.

### 3.2. Comparison of the Six Different Cuttings Types of “Bither” Cultivar

Results of the descriptive data analysis recorded high morphological variation in the plant material derived from the 6 fig cutting types of the cultivar “Bither,” for all the traits. The most widely varied parameters were root emergence percentage (0–90%), length of nursery plant (3–77 cm), and number of roots/nursery plant (0–29 roots) (Table 4).

Results of 6 cutting types for the cultivar “Bither” analysis showed a significant difference at a level of 5% in all the studied characters (Table 5). The length of nursery plant (LP), diameter of nursery plant (DP), cumulative growth of nursery plant (CG), and number of roots/nursery plant (NR) showed high significant difference. Results revealed that root emergence percentage (RE %) had the highest value in cutting type 3 and the lowest value in type 6. Also, type 3 exhibits the highest means of number of roots/nursery plant (NR), length of nursery plant (LP), and number of leaves/nursery plant (NL). The cutting type 5 was significantly high in the cumulative growth trait in nursery plant (CG). Finally, the number of roots/nursery plant (NR) presents the highest value in cutting type 5.

The findings of the study put forth that cutting characteristics (length, diameter, and age) affected the growth and quality of fig plants. Moreover, cutting length affected the length (LP) and cumulative growth (CG) of fig nursery plant. The use of long cuttings (40–60 cm) is recommended to produce vigorous fig nursery plants.

## 4. Discussion and Conclusions

The results achieved in this study showed that propagation of fig plant material under field conditions can be applied and should not require sophisticated equipment [6, 12]. The values of characters measured on nursery fig plants in the present study were compared with those from other countries, namely, Morocco [12, 15], Turkey [14, 16], Iran [13], Thailand [17], and Chile [18]. Similar percentage of rooted cuttings on some Moroccan varieties propagated by using hardwood stem cutting under field conditions was found. “Chaari,” “Aqounaq El Hmam,” “Ghouddane,” “Ournaki,” and Kadota presented high values of % root emergence; 89, 98, 94, 87, and 88%, respectively [12]. Cultivar “Sarilop” from Turkey has revealed high similarity in morphological characteristics of nursery plants with “Bayoudhi” and “Jemaâoui” cultivars from the present study [14]. In Turkey, the fig cultivar Calimyrna “Sarilop” is used and propagated extensively by cuttings and “Calimyrna” is the most popular and best cultivar in the world because it dries well. Nevertheless, rooting ratio %, length of nursery plant, and diameter of nursery plant reported lower values than those of Tunisian cultivars studied [14]. The Iranian cultivar “Sabz” represents the large majority of fig trees present in Estahban area. Obtained values on root emergence percentage, number of emergence buds, length of nursery plant, and number of leaves/nursery plant variables are similar to our results [13]. “Brown Turkey,” “Dauphine,” and “Lisa” cultivars from Thailand showed variables values of nursery fig plants comparable with those of this work. However, root emergence percentage revealed lower values (12.50–39.29%) than that found in the present survey (15–83.33%), whereas three cultivars showed higher values for the number of emerged buds and number of roots/nursery plant [17].

Moreover, the high variation was recorded among the % roots emergences and the other morphological traits within the 5 cultivars (“Bayoudhi,” “Jemâaoui,” “Ragoubi,” “Zidi,” and “Bouharrag”) and between the 6 different types of cuttings (age, length, and diameters) of the cultivar “Bither”. These variations were influenced by ability of cultivar for rooting and characteristics of cuttings (length, diameter, and age) [14]. Furthermore, practices of farming (soil preparation, irrigation, harrowing, etc.) and climate conditions were cited to have an effect on the quality of fig plants in the nursery [13, 18].

Several factors seem to be involved in the improvement of fig hardwood cutting like the use of rooting hormonal treatment [15] and the use of plastic tunnels in fig propagation [17]. In fact, these trails are under study in our laboratory.

The present research showed that the hardwood cuttings of local cultivars of figs can be used to propagate material in field conditions. Furthermore, it is possible to obtain vigorous young plants (Length, number of leaves, and cumulative growth) from cuttings of cultivars “Bouharrag” (Caprifig), “Bayoudhi” (Common fig), “Rogabi,” and “Zidi” (Smyrna fig). “Bither's” rooting ability of the 6 cutting types studied revealed that the cutting type of one-year old characterized by 60 cm of length and diameter of <1.5 cm gave the best percentage of rooting emergence. Also, the best cumulative growth of plant was obtained with the cutting type (2 years old shoots, length (40 cm), and diameter (<1.5 cm)).

The positive aspects of this experiment have demonstrated the suitability of the hardwood cuttings method for vegetative propagation, since the production of fig plants in nursery can be realized and seems to be cheap. This work is supposed to be a preliminary positive contribution in the propagation of fig in nursery which can have practical application for fig horticulturists in the southeast of Tunisia.

## Figures and Tables

**Table 1:** Fig hardwood cuttings experiments in split-plot design.

Experiment	Cultivar	Replicate 1	Replicate 2	Replicate 3
Experiment 1	“Bouharrag”	10 cuttings	10 cuttings	10 cuttings
	“Bayoudhi”	10 cuttings	10 cuttings	10 cuttings
	“Jemaâoui”	10 cuttings	10 cuttings	10 cuttings
	“Rogabi”	10 cuttings	10 cuttings	10 cuttings
	“Zidi”	10 cuttings	10 cuttings	10 cuttings

Experiment 2	“Bither”: cutting type 1	10 cuttings	10 cuttings	10 cuttings
	“Bither”: cutting type 2	10 cuttings	10 cuttings	10 cuttings
	“Bither”: cutting type 3	10 cuttings	10 cuttings	10 cuttings
	“Bither”: cutting type 4	10 cuttings	10 cuttings	10 cuttings
	“Bither”: cutting type 5	10 cuttings	10 cuttings	10 cuttings
	“Bither”: cutting type 6	10 cuttings	10 cuttings	10 cuttings

**Table 2:** Mean, standard deviation, and range of morphological characters recorded in nursery plants derived from cuttings for five fig cultivars.

Characters	Mean ± standard error	Standard deviation	Range
% Root emergence (RE %)	48.00 ± 7.86	24.85	10–90
Number of emerged buds/nursery plant (NB)	0.54 ± 0.13	0.89	0–3
Length of nursery plant (LP cm)	28.08 ± 2.23	15.47	4–70
Diameter of nursery plant (DP mm)	11.96 ± 0.92	6.43	5–45
Number of leaves/nursery plant (NL)	18.96 ± 1.77	12.29	3–63
Cumulative growth of nursery plant (CG cm)	60.44 ± 2.60	18.03	32–112
Number of roots/nursery plant (NR)	15.02 ± 1.29	8.93	1–39

**Table 3:** Means of nursery plants morphological characters studied in five fig cultivars (means of two replications 2009 and 2010).

Cultivar	RE %	NB	LP cm	DP mm	NL	CG cm	NR
Bouharrag	40.00^b^	1.25^b^	40.50^b^	14.75^b^	37.13^c^	73.50^b^	19.25^b^
Bayoudhi	15.00^a^	0.67^ab^	62.00^c^	24.33^c^	27.00^b^	101.00^c^	28.33^c^
Jemaâoui	65.00^c^	0.85^ab^	21.77^a^	9.08^ab^	14.23^a^	53.62^a^	13.23^ab^
Rogabi	65.00^c^	0.15^a^	20.85^a^	8.38^a^	14.69^a^	53.92^a^	9.85^a^
Zidi	55.00^bc^	0.09^a^	25.82^a^	14.18^b^	14.18^a^	55.64^a^	16.55^ab^

*F* calculated	2.225	3.572	12.022	8.325	11.134	11.944	4.397

Significance	∗∗	∗	∗∗	∗∗	∗∗	∗∗	∗∗

∗, ∗∗: significant, high significant at *P* < 0.05, respectively.

Different letters in one column indicate differences within cultivars by least significant difference (LSD) test at a significance level of 0.05.

**Table 4:** Mean, standard deviation, and range of morphological characters recorded in fig cutting types in nursery.

Characters	Mean ± standard error	Standard deviation	Range
% Root emergence (RE %)	58.38 ± 4.82	22.12	0–90
Number of emerged buds/nursery plant (NB)	0.72 ± 0.12	1.30	0–9
Length of nursery plant (LP cm)	23.96 ± 1.66	17.97	3–77
Diameter of nursery plant (DP mm)	10.80 ± 0.50	5.41	3–32
Number of leaves/nursery plant (NL)	16.79 ± 0.96	10.40	2–62
Cumulative growth of nursery plant (CG cm)	63.51 ± 1.78	18.77	31–105
Number of roots/nursery plant (NR)	9.46 ± 0.60	6.33	0–29

**Table 5:** Means of nursery plants morphological characters studied in six cuttings types of Bither's fig cultivar (means of two replications 2009 and 2010).

Cutting types	RE %	NB	LP cm	DP mm	NL	CG cm	NR
Type 1	63.33^b^	0.58^ab^	19.11^a^	8.79^a^	14.63^a^	53.29^a^	4.76^a^
Type 2	63.33^b^	0.21^a^	21.58^a^	10.16^a^	13.58^a^	55.21^a^	10.89^bc^
Type 3	83.33^b^	0.56^ab^	24.52^a^	9.68^a^	16.04^ab^	65.04^abc^	10.50^bc^
Type 4	53.33^ab^	0.44^ab^	18.19^a^	11.81^a^	13.75^a^	58.29^ab^	14.75^c^
Type 5	50.00^ab^	0.73^ab^	24.13^a^	10.53^a^	17.07^ab^	77.20^c^	9.53^b^
Type 6	26.67^a^	1.38^bc^	18.25^a^	9.75^a^	22.50^bc^	69.38^bc^	6.38^ab^

*F* calculated	3.166	2.784	3.370	3.294	2.784	4.204	4.232

Significance	∗	∗	∗∗	∗∗	∗	∗∗	∗∗

Type 1: type cutting of one-year-old shoots, length (30 cm), and diameter (<1.5 cm).

Type 2: type cutting of one-year-old shoots, length (40 cm), and diameter (<1.5 cm).

Type 3: type cutting of one-year-old shoots, length (60 cm), and diameter (<1.5 cm).

Type 4: type cutting of 2-year-old shoots, length (30 cm), and diameter (<1.5 cm).

Type 5: type cutting of 2-year-old shoots, length (40 cm), and diameter (<1.5 cm).

Type 6: type cutting of 2-year-old shoots, length (60 cm), and diameter (>2 cm).

∗, ∗∗: significant, high significant at *P* < 0.05, respectively.

Different letters in one column indicate differences within cutting types by least significant difference (LSD) test at a significance level of 0.05.
